# Molecular Study of the Amazonian Macabea Cattle History

**DOI:** 10.1371/journal.pone.0165398

**Published:** 2016-10-24

**Authors:** Julio Vargas, Vincenzo Landi, Amparo Martínez, Mayra Gómez, María Esperanza Camacho, Luz Ángela Álvarez, Lenin Aguirre, Juan Vicente Delgado

**Affiliations:** 1 Universidad Nacional Amazónica. Rectoría. Puyo, Ecuador; 2 Departamento de Genética, Universidad de Córdoba, Córdoba, España; 3 Animal Breeding Consulting S.L., Parque Científico Tecnológico de Córdoba, España; 4 Instituto de Investigación y Formación Agraria y Pesquera (IFAPA), Alameda del Obispo, Córdoba, España; 5 Universidad Nacional de Colombia - Sede Palmira, Palmira Colombia; 6 Universidad Nacional de Loja, Loja, Ecuador; Senckenberg am Meer Deutsches Zentrum fur Marine Biodiversitatsforschung, GERMANY

## Abstract

Macabea cattle are the only *Bos taurus* breed that have adapted to the wet tropical conditions of the Amazon. This breed has integrated into the culture of the indigenous Shuar-Asuar nations probably since its origins, being one of the few European zoogenetic resources assimilated by the deep-jungle Amazon communities. Despite its potential for local endogenous sustainable development, this breed is currently endangered. The present study used molecular genetics tools to investigate the within- and between-breeds diversity, in order to characterize the breed population, define its associations with other breeds, and infer its origin and evolution. The within-breed genetic diversity showed high values, as indicated by all genetic parameters, such as the mean number of alleles (MNA = 7.25±2.03), the observed heterozygosity (Ho = 0.72±0.02) and the expected heterozygosity (He = 0.72±0.02). The between-breeds diversity analysis, which included factorial correspondence analysis, Reynolds genetic distance, neighbor-joining analysis, and genetic structure analysis, showed that the Macabea breed belongs to the group of the American Creoles, with a Southern-Spain origin. Our outcomes demonstrated that the Macabea breed has a high level of purity and null influences of exotic cosmopolitan breeds with European or Asiatic origin. This breed is an important zoogenetic resource of Ecuador, with relevant and unique attributes; therefore, there is an urgent need to develop conservation strategies for the Macabea breed.

## Introduction

The group of the American Creole cattle breeds, which were formed in the American continent after the European colonization [1], represents an important economic resource for marginal areas of South America; however, these breeds are still not well characterized. Macabea cattle, named after the Macas Province in Ecuador, is a singular breed adapted to the wet tropic of the Amazon, integrated as a patrimony of the indigenous nationalities.

The first arrival of cattle to America occurred in the second voyage of Columbus in 1493, which set sail from the south of the Iberian Peninsula [2]. After this first, other sporadic introductions were carried out by the Spanish governorate and in the Portuguese colonies, through the Brazilian *Capitanies* [3]. All the Spanish and Portuguese breeds have a Bos Taurus origin. After the independence of the American colonies in the late 19th century, the British economic influence enabled the progressive introduction of *Bos indicus* breeds from the Asian colonies of the British Empire. Already at the beginning of the 20th century, Asiatic breeds such as Guzerat, Nelore, and Gyr had an extended distribution across the United States, Mexico, and Brazil [4].

*Bos taurus* and *B*. *indicus* have a common ancestor in the Aurochsen wild *B*. *primigenius*, which marked the start of domestication approximately 11000 years ago [5], even though it commenced from two different domestication processes and geographical locations [6].

The Amazonian region was almost free of cattle until the 20th century, when farming practices became more aggressive and sprawled into natural environments. At the same time, the introduction of *Bos indicus* animals became massive and indiscriminate, producing extensive deforestation especially in river basins. Today only few local breeds from *Bos Taurus* type exist one of this is the Ecuadorian Macabea breed. The animals from this breed present excellent meat attributes.

The origin of the Macabea breed is unknown. Presumably, they reached the Ecuadorian Amazon along with the Spanish incursions developed between 1540 and 1548 from Quito and Guayaquil [7]. However, European practices were not consolidated in the region until 1576, with the foundation of the Sevilla de Oro city, which was destroyed in 1599 by the Shuar-Achuar native nations [7]. Probably during this period, native communities assimilated some European resources, such as the precursors of the current Macabea breed.

**Fig 1:**
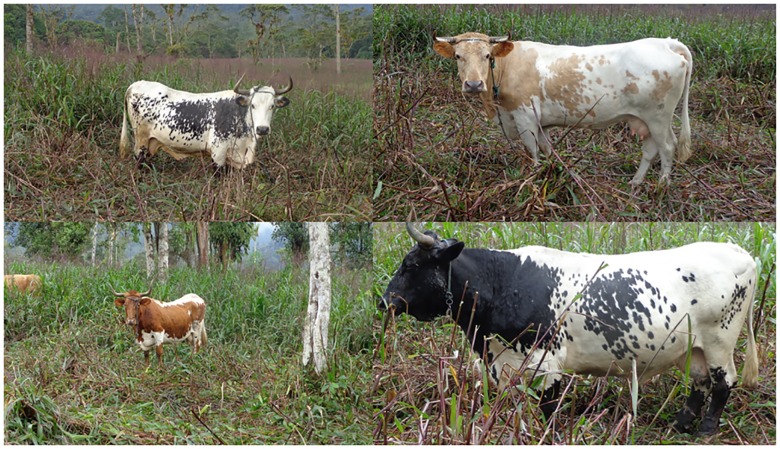
Four of the Macabea bovines included in this study.

Macabea cattle (Fig 1) are currently distributed in small herds composed by 1 to 10 individuals. The total number of herds and total number of individuals are unknown, but official reports classify this breed as in critical situation according to the FAO´s criteria [8].

There are several enigmatic controversies around the genetics origins of Macabea breed, and in particular, if it should be admitted as a resource for the sustainable development of the Amazonian region. There is a persistent pressure from conservationist groups against the presence of farm animals in the natural environment. The aim of this study was to genetically characterize the Macabea cattle breed based on microsatellites markers, in order to generate scientific data about this rare genetic resource and its genetic relation with other Creole or exotic breeds, searching for a geo-evolutionary explanation of its origin and dissemination. These findings would support the conservation of this important breed, and propose the Macabea breed as a resource for the endogenous sustainable development of the region, in terms of its excellent meat quality and its ability to adapt to the wet tropic. Microsatellite markers have been widely used for population genetic analyses of livestock species [9–11], as they are informative and can successfully elucidate associations between individuals and populations, introgression from other populations, genetic differentiation, and admixtures between breeds [12–14].

## Materials and Methods

### Ethics Statement

Ethical approval was not needed for this study. All hair samples collections were non-invasive (hair roots) and carried out during routine veterinary visits in the farms on live animals. The fieldwork did not involve any endangered or protected species. Hair root were manually collected without any injury in the back of the animals No other kind of tissues (blood, meat or other) were used in this study.

### Sampling and DNA extraction

Hair samples of 25 Macabea individuals (Fig 1)were randomly collected from eight herds (3–4 animal per herd and when applicable one male and 2 female) distributed in the 55,280 km^2^ area of the Amazonian Provinces of Pastaza and Morona Santiago, in Ecuador; in particular the following location were visited: Palora (long: -77.975505, lat: -1.720847°); Jimbitono (long: -78.177398°, lat: -2.260553°); Macas (long: -78.106919°, lat: -2.318572°); Puyo (long: -78.046440°, lat: -1.530083°); Morona-Santiago (long: -78.289073°, lat: -2.556032°); All the farms were isolated, distributed in the deep-jungle, accessed only by river routes, and with a considerable distance between them (~50–100 km). Cattle owners belonged to the native nationality Shuar-Achuar, which still maintain strong traditional culture practices. After collection, hair samples were stored in labeled envelopes, and brought from the collecting point to the Universidad Estatal Amazónica (Puyo, Ecuador). DNA was extracted using three hair roots cut and incubated at 95°C during 15 minutes and at 99°C during 3 minutes in a 100 μl solution of Chelex100 resin (Bio-Rad, California, USA). After incubation, extracted DNA was conserved at –20°C until use.

### Genotype analysis

A panel of 28 microsatellite markers (Table 1), selected on the basis of the recommendations issued by the FAO/International Society of Animal Genetics, were used to conduct studies on bovine genetic biodiversity [15]. The analysis was performed in the Laboratory of Applied Molecular Genetics of the Research Group PAI-AGR-218, in the University of Córdoba (Spain).

**Table 1:** Outcomes of the Microsatellite Analysis.

Microsatellite	NA	He	Ho	PIC	*F*_IS_	*P*-value
**BM1314**	6	0.731	0.680	0.671	0.072	0.250
**BM1818**	6	0.477	0.520	0.425	-0.093	0.437
**BM1824**	4	0.666	0.600	0.600	0.101	0.084
**BM2113**	8	0.839	0.720	0.801	0.145*	0.020*
**BM8125**	5	0.578	0.640	0.527	-0.110	0.598
**CRSM60**	8	0.739	0.760	0.694	-0.029	0.670
**CSSM66**	10	0.898	1.000	0.866	-0.117	0.073
**ETH003**	8	0.851	0.920	0.813	-0.082	0.379
**ETH010**	7	0.746	0.800	0.691	-0.074	0.686
**ETH185**	6	0.456	0.440	0.410	0.035	0.0130*
**ETH225**	7	0.774	0.800	0.720	-0.035	0.663
**HAUT24**	7	0.753	0.720	0.699	0.044	0.417
**HAUT27**	10	0.810	0.800	0.769	0.012	0.253
**HEL09**	8	0.790	0.800	0.741	-0.013	0.779
**HEL13**	5	0.659	0.760	0.607	-0.157	0.311
**ILSTS006**	9	0.839	0.909	0.798	-0.085	0.156
**ILSTS011**	6	0.734	0.680	0.678	0.075	0.274
**INRA023**	8	0.813	0.880	0.769	-0.084	0.852
**INRA032**	7	0.740	0.680	0.689	0.082	0.493
**INRA035**	4	0.602	0.280	0.503	0.540*	0.00*
**INRA037**	9	0.771	0.667	0.719	0.138	0.275
**INRA063**	5	0.654	0.560	0.576	0.146	0.096
**MM12**	8	0.738	0.800	0.679	-0.086	0.075
**SPS115**	6	0.614	0.840	0.558	-0.379*	0.284
**TGLA053**	8	0.684	0.520	0.629	0.244*	0.007*
**TGLA122**	12	0.848	0.800	0.812	0.058	0.408
**TGLA126**	5	0.735	0.667	0.666	0.095	0.927
**TGLA227**	11	0.855	0.880	0.819	-0.030	0.925
Mean	7.3	0.728	0.719	0.676	0.015	0.372

* Significance value (*P*<0.05);

NA: number of alleles detected; He: unbiased expected heterozygosities; Ho: observed heterozygosities (Ho); PIC: content of polymorphic information; *P*-value: probability values obtained in the Hardy-Weinberg equilibrium test.

Data used in this paper have been archived at Dryad (www.datadryad.org): doi:10.5061/dryad.1dh4c

We obtained data on different bovine breeds from the BIOBOVIS project (http://www.biobovis.jimdo.com); these breeds represented evolutionary branches to be tested to determine the genetic origin and the evolutionary associations of Macabea. Data are deposited and available at Laboratory of Applied Molecular Genetics. As outgroup-breeds, we chose the Rubia Gallega (RGA), which represents Celtic animals from northern Spain, and the Berrenda en Colorado (BC) and the Marismeña (MA), both representing southern Spanish resources. In theory, Macabea cattle reached the Amazon with colonizers from the Pacific coast; therefore, we included the Ecuadorian Creole Ecuadorian of Southern (EC) and the Colombian Creoles Hartón del Valle (HV), and Blanco Orejinegro (BON). In order to test recent influences of cosmopolitan European breeds specialized in milk and beef production, we included the most influential breeds of these groups in the region: Hereford (HER), Brown Swiss (BWS), Holstein (HOL), Jersey (JER), Simmental (SIM), and Charolaise (CHAR). Finally, to test the possible influence of the zebuine expansion in the Amazon on the Macabea breed, the Brahman (BRH), Gyr (GYR), Nellore (NEL), and Cuban Zebu (ZEBU) were also used in this study. To explore the between-breeds diversity, we used 621 genotypes belonging to outgroup-animals. This information, and the individual breeds sample sizes are shown in Table 2.

**Table 2:** Biodiversity Parameters of the 17 Analyzed Breeds.

Population	SZ	NA	AR	He	Ho	*F*_IS_	HW
BON	25	5.74±1.76	5.28	0.70±0.02	0.74±0.02	-0.06	1
HV	22	7.74±1.73	7.24	0.78±0.02	0.78±0.02	0.00	2
EC	58	9.47±2.44	7.64	0.79±0.02	0.72±0.01	0.09*	4
RGA	50	7.47±1.95	6.12	0.71±0.03	0.70±0.01	0.02	1
BC	40	7.68±2.16	6.76	0.78±0.02	0.73±0.02	0.06*	1
MA	50	7.79±2.55	6.19	0.74±0.02	0.72±0.01	0.02	1
HER	88	6.63±1.54	5.27	0.70±0.02	0.65±0.01	0.07*	2
JER	20	4.79±1.03	4.57	0.65±0.03	0.67±0.02	-0.03	1
BWS	29	6.79±2.15	6.10	0.73±0.02	0.74±0.02	-0.02	2
CHAR	58	6.95±1.99	5.79	0.71±0.03	0.68±0.01	0.03	1
FRI	50	6.89±2.40	5.82	0.71±0.03	0.73±0.01	-0.02	1
SIM	19	6.16±1.92	5.88	0.67±0.03	0.65±0.03	0.03	2
GYR	36	7.00±1.94	5.97	0.67±0.03	0.62±0.02	0.07*	4
BRH	41	7.74±2.40	6.33	0.70±0.02	0.68±0.02	0.03	3
NEL	49	6.89±1.94	5.41	0.63±0.02	0.59±0.02	0.07*	7
ZEBU	50	7.53±2.06	6.28	0.71±0.04	0.71±0.01	-0.00	4
MAC	25	7.79±2.02	6.92	0.73±0.03	0.73±0.02	0.01	3
Mean	42	7.13±2.00	6.23	0.70±0.02	0.74±0.02	0.03*	

*: Significant value (*P*<0.05);

SZ: sample size; NA: total number of alleles; AR: allelic richness (considering 16 samples); He: expected heterozygosity; Ho: observed heterozygosity; *F*_IS_: fixation index within population; HW, Hardy-Weinberg equilibrium (deviated loci per breed; *P*<0.05); BON: Blanco Orejinegro; HV: Hartón del Valle; EC: Ecuadorian Creole Ecuadorian of Southern; RGA: Rubia Gallega; BC: Berrenda en Colorado; MA: Marismeña; HER: Hereford; JER: Jersey; BWS: Brown Swiss; CHAR: Charolais; FRI: Holstein Friesian; SIM: Simmental; ZEBU, Zebu; NE: Nelore; BRH: Brahman; GYR: Gyr; MAC: Macabea.

### Statistical analysis

#### Within-breed diversity

To explore the within-breed genetic diversity in the Macabea, we calculated allelic frequencies, observed heterozygosity (Ho), unbiased expected heterozygosity (He), the average number of alleles (Na) and the content of polymorphic information (PIC) for the breed and for each marker by means of using the Microsatellite Toolkit software [16]. The Fis coefficient for Macabea (Weir and Cockerham, 1984) was calculated using the software Genetix v.4.05.2 [17]. The Hardy-Weinberg(HW) equilibrium test was performed using the Genepop software v. 4.2 [18], which apply the Fisher-exact-test based on the Markov chain Monte Carlo method [19].

#### Between-breed diversity

To infer the genetic relation between the Macabea breed and all the determined outgroups, we performed a factorial correspondence analysis (FCA) using the Genetix v. 4.05.2 software [17]. In addition, the pair-wise Fst and the Reynolds genetic distances between populations were calculated [20] by means of using Populations v.1.2.28 software [21]. The calculation and graphical representation of the Fst matrix were performed using the Arlequin software v.3.5[22]. Based on the obtained genetic distance-matrix, we constructed a neighbor-joining dendrogram (neighbor-net) with SplitsTree v. 4.0 software [23]. The genetic structure of the populations included in this study was explored using the Structure v. 2.3.4 software [24]. The parameters used were 200K iteration after 100K burn-in under the Admixture model with default settings. This program uses a Bayesian algorithm to calculate *a posteriori* distribution of each individual admixture coefficient (q). The mean in this distribution represents an estimation of the proportion of the parental population genome present in the individuals. This program develops a clustering of the individuals in different number of clusters (K), representing the number of populations admitted in an admixture model, in which each individual genome could content different percentages of the ancestral population genomes where they come from. Alternatively, two structure runs were carried out: in the first one, all breeds were included, and the results were displayed using Distruct v1.1 software [25] and the most likely numbers of group (K) were assessed by the Evano method [26]. In the graphical representation, each individual is represented by a vertical line divided in *k* colored segments, which represents the genotypic fractions of each inferred cluster. Secondly, we calculated a structure run only including MAC, EC, BON and HV breeds, where we used the kriging interpolation method [27] to assess the correlation between assignment values and geographical data. After this, the graphical library of statistical software R version 3.2.4[28] was used to display the maps, where each breed was represented by coordinates corresponding to the center of their geographical dispersion.

## Results

### Microsatellite markers

The microsatellites panel used in this study has been previously applied and proved in several cattle studies, conducted by our own research team [1, 2, 29]. This panel allowed the detection of 316 alleles, with a mean of 7.3±2 alleles/locus, with a global observed and expected heterozygosities of 0.72 and 0.73, respectively. The most polymorphic marker in terms of number of alleles was TGLA122, with a value of 12; meanwhile, the lesser polymorphic markers were BM1824 and INRA035 (4), both with a value of four. The expected heterozygosities by marker were high (Table 1), and ranged from a minimum of 0.456 (ETH185) to a maximum of 0.898 (CSSM66). The HW equilibrium was generally respected; in fact, only four markers (BM2113, ETH18, INRA035, TGLA053) presented significant deviations (Table 1).

### Breed diversity

Most of the markers in the studied breeds were in HW equilibrium (Table 2); seven breeds showed only one marker out of the equilibrium, four breeds only two markers deviated, two breeds three, and three breeds showed four markers out of the equilibrium. Only Nellore breed presented a higher number of unbalanced markers, with seven. Overall, a mean of 2.35 markers deviated from the HW equilibrium. Macabea showed three deviated markers, slightly above the mean. The average F-statistics and their 95% confidence intervals (data not shown) obtained with 10,000 bootstraps over loci were: *F*_IS_ = 0.03(0.02–0.05), *F*_IT_ = 0.16(0.14–0.18), and *F*_ST_ = 0.13(0.12–0.15). Macabea cattle showed a high mean of alleles, with a value 7.79±2.02; the other Ecuadorian breed EC presented the highest value (9.47±2.44), and the lower value was detected in JER (4.79±1.03). This trend was confirmed by the allelic richness and the heterozygosity data (Table 2). The overall defect of heterozygous estimated by mean the *F*_IS_ index was low in all dataset (0.03) and significant (*P*<0.05). When observed by breed, the *F*_IS_ index from EC, BC, HER, GYR, and NEL breeds showed slightly significant values, and with Macabea presented no significant deviation.

### Between-breeds associations

**Fig 2:**
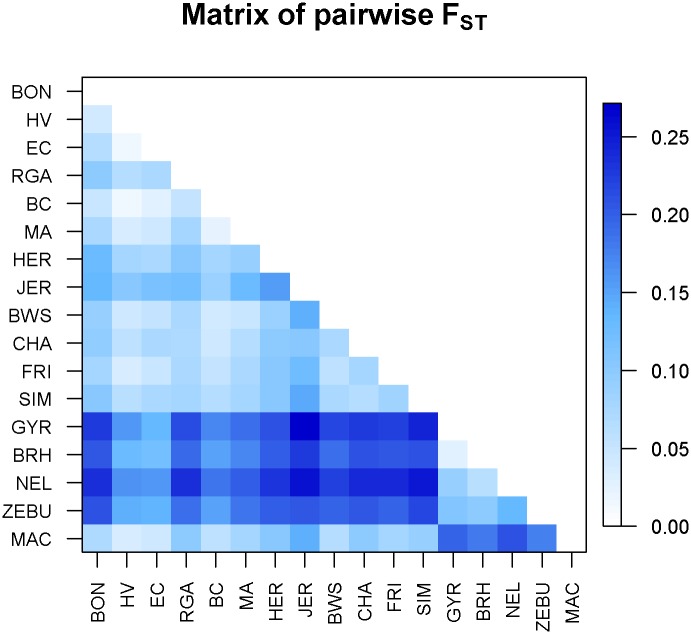
Pairwise *F*_ST_ distance matrix graphical representation.

S1 Table presents the results of the two genetic distances between the studied breeds explored (*F*_IS_ and Reynolds distances). In both cases, Macabea showed extreme distances in respect to the zebuine breeds, and a close position in respect to the EC and the Colombian breeds (HV and BON). These results are supported by Fig 2, where *F*_ST_ pairwise-distances are graphically represented by using a color gradient matrix. Two clusters belonging to the *Bos indicus* and *Bos taurus* cattle types are visible. In this scenario, Macabea cattle breed showed very large distances with respect to all the *Bos indicus* breeds, with *F*_ST_ values ranging between 0.18 and 0.23, and between 0.18 and 0.21 for Reynolds distances. In general, Macabea breed also showed important distances in respect the other international *Bos taurus* breeds, with *F*_ST_ values ranging between 0.07 and 0.15, and between 0.06 and 0.14 for Reynolds distances. The shorter distance of Macabea with international breeds was detected in respect to the BWS breed. The Spanish outgroups RGA (representing the Celtic breeds from the northern Spain), and the MA and BC (Southern-Spain breeds) showed diverse patterns of distances with MAC, being the southern breeds closer than the northern breeds, with *F*_ST_ values of 0.06, 0.08, and 0.11, respectively, and Reynolds distances of 0.06, 0.08 and 0.10, respectively. Finally, the group that was detected to be the most related to Macabea was formed by the other Creoles, in particular, the Colombian HV and the EC (both at FST 0.04; 0.04 and 0.036 of Reynolds distance respectively).

**Fig 3:**
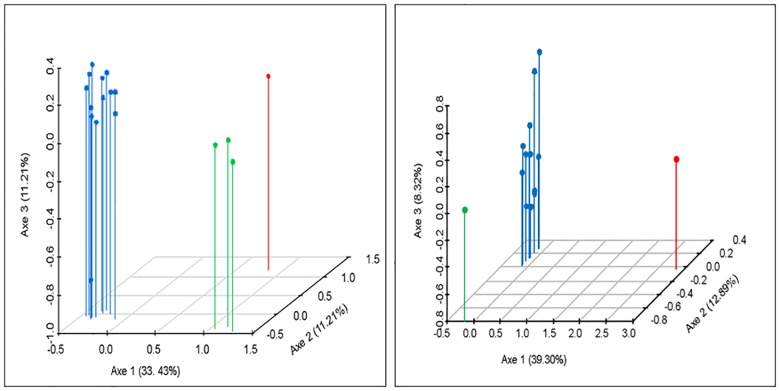
Graphic representation of the factorial correspondence analysis results. a) Factorial correspondence analysis results involving the 17 cattle breed populations; and b) factorial correspondence analysis results without *Bos indicus* breeds. In a) Red: ZEBU; Green: GYR. BRH. NEL; Blue: BON, HV. EC, RGA. BC, MA, HER, JER, BWS, CHAR, FRI, SIM, MAC; in b) Red: MAC; Green: HER; Blue: BON, HV, EC, RGA, BC, MA, JER, BWS, CHAR, FRI, SIM.

**Fig 4:**
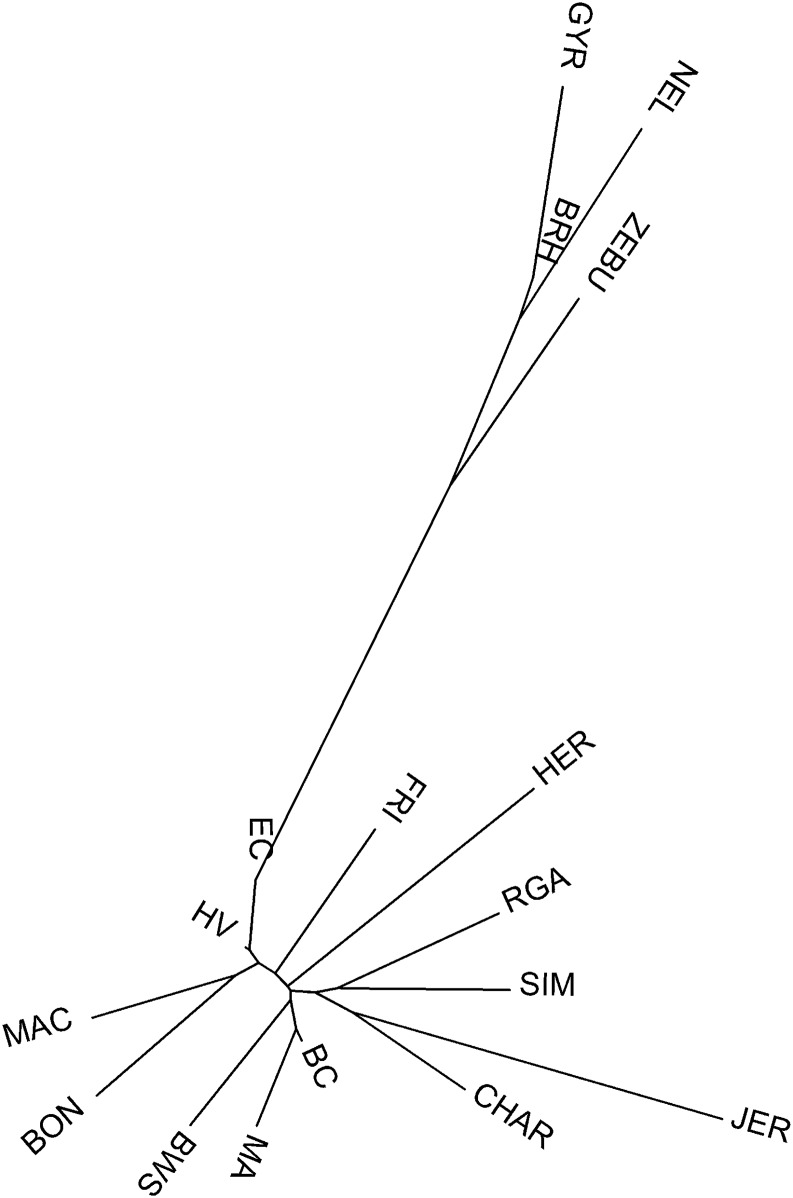
Neighbor-joining dendrogram constructed from the Reynolds genetic distances between 17 cattle breeds. EC: Ecuadorian Creole Ecuadorian of Southern; HV: Hartón del Valle; FRI: Holstein Friesian; BON: Blanco Orejinegro; MA: Marismeña; BC: Berrendaen Colorado; BWS: Brown Swiss; SIM: Simmental; HER: Herford; CHAR: Charolais; JER: Jersey; RGA: Rubia Gallega; MAC: Macabea; ZE: Zebu; NEL: Nellore; BRH: Brahman; GYR: Gyr.

Analysis of molecular variance [22, 30, 31] were performed considering two levels of classification; the first one, grouped by breed branch (*Bos taurus* vs. *Bos indicus*); and the second level considered each geographical origin (i.e., *Bos indicus* and international breeds were considered as two separate groups). Table 3 shows that the highest percentage of variation was always within-breeds, with a value of 80.03% and 83.90% for phylogenetic and geographical groups comparisons, respectively (*P*<0.01). Variation between-groups were 11.67% (*P* <0.05) and 7.86% (*P*<0.01) for the phylogenetic and geographical groups comparisons, respectively. The results of the factorial correspondence analysis conducted with all the studied breeds, and then avoiding the zebuines, both supported the results obtained in the AMOVA: breeds were grouped in the space according to their phylogenetic origin (Fig 3). The distance tree (Fig 4), constructed with the individual Reynolds distances by using the neighbor-joining algorithm, also showed congruent results with respect to the geographic and phylogenetic origins: all zebuine breeds occupied similar distance-branch, and Macabea was close to the other Creoles, particularly with HV. There was no relevant influence from other international *Bos taurus* or *Bos indicus* breeds on Macabea.

**Table 3:** Outcomes from the Analysis of Molecular Variance: results are expressed as a percentage of explained variance and resulting fixation indices, comparing within- and between-breeds according to their phylogenetic and geographical origins.

	factor considered	
	phylogenetic	geography
sum of square/ degree of freedom		
among groups	537.214/1	654.406/3
**AMONGBREEDSWITHINGROUP**	849.924/15	732.732/13
**WITHINBREED**	8642.129/1403	8642.129/1403
variance components		
among groups	0.90	0.60
**AMONGBREEDSWITHINGROUP**	0.61	0.60
**WITHINBREED**	6.15	6.16
percentage variation		
among groups	11.67**	7.86***
**AMONGBREEDSWITHINGROUP**	8.03***	8.24***
**WITHINBREED**	80.30***	83.90***
f-statistics		
**AMONGGROUP (FCT)**	0.20	0.16
among breeds within group (fsc)	0.10	0.10
among breeds total variability (fst)	0.12	0.10

***: *P*-value<0.001,

**: *P*-value<0.005

**Fig 5:**
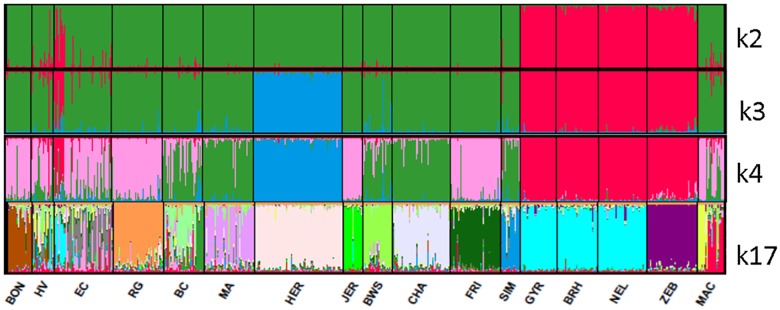
Graphic representation of the genetic structure analysis of the 17 cattle breeds. In each K graphic, the color represents the genetics partition found by the software.

Neighbor-joining dendrogram (Fig 4) based on Reynolds distances between the 17 studied breeds, showed large distances between zebuins and taurines. Macabea formed a cluster with its Colombian neighbor BON, proximal to the other regional Creoles HV and EC. However, Fig 4 does not clearly define which breed group (Spanish or international ones) has more influenced the origin of Macabea. Results of the genetic structure analysis represented in Fig 5 are eloquent in respect to the definition of the breeds. From k2, the differentiation between zebuines and taurines is evident; from k3, the structure of the different breeds is beginning to be definite; and in k17, all the structures of the studied breeds are evident. The most likely K value as indicated by the Evano method was K = 14 even though a higher peak was visible at K = 3 (S1 Fig).

**Fig 6:**
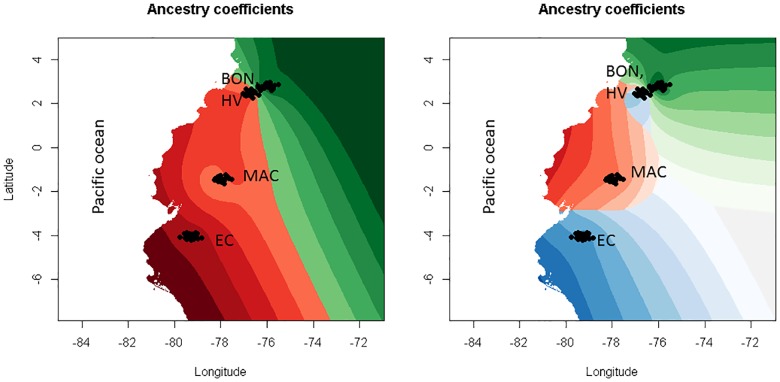
Geographical representation of the interpolation of admixture coefficients (*Q* matrix) for clusters k2 and k3 including MAC, EC BON and HV breeds only. Each color represents the genetics partitions spotted on geographical surface through Kriging algorithm.

Geographical representation of the interpolation of the admixture coefficient (Q matrix), using as reference the clusters k2 and k3, results very illustrative. Fig 6 shows the close relation of Macabea and its equidistant position in respect to the pacific Colombian Creoles (BON and HV) in the north, and with the EC in the south.

## Discussion

We described a unique cattle breed adapted in a tropical environment of Ecuador. The importance of this genetic resource goes further in the field of biodiversity conservation; in fact, these animals reared in extremely marginal areas, with low technological inputs, demonstrate a unique capacity of adaptation to produce high quality beef in the wet tropics, intended as a money reservoir for native nationalities. In general, the daily protein consumption of families is sustained through hunting, fishing, and pigs and chicken. The cattle, however, are often converted into money when needed by the family. The official recognition supported by the molecular findings could add value to this particular economy and improve the quality of life of the disadvantaged population.

Within-breed diversity results supplied robust and important arguments to support Macabea conservation status. We must first highlight that only four of our 28 markers were not in HW equilibrium; this outcome denotes a genetic stability in the population that corresponds to a consolidated breed with no recent bottleneck or migration events. Other general parameters also support this hypothesis, such as the mean number of alleles, all of which reflect a high level of within-breed variability. These general parameters were higher than those previously reported for European breeds [11, 14, 32, 33], but were similar to values previously described for other Latin American breeds [34, 35].

Cattle reached Ecuador from Panama during the beginning of the regional Spanish conquest arriving directly to the Caribbean Islands, which was the first point of cattle colonization in the Americas [36]. Probably, the first introduction of cattle to the Amazon derived from the Pacific coast, between the years of 1540 and 1548 [7], along with the Spanish conquest and colonization.

The panel of microsatellites markers used in the present study resulted highly informative and with sufficient statistical power for biodiversity studies as just described in different works on bovine species [2] suitable as showed by high allelic richness, and PIC levels (Table 1). Therefore, our panel is recommended for any other research on Creole biodiversity studies, confirming previous findings of our own team developed with American Creole cattle [1].

The general *F*_IS_ values, which were undistinguishable from cero, demonstrated a general HW equilibrium. These results indicated that Macabea presents a level of genetic differentiation that is sufficient to be considered as an independent breed, according to international conventions [37]. Thus, Macabea is revealed as an important genetic resource belonging to the Ecuadorian patrimony, and further efforts should be made to recognize, protect, and promote this breed.

Once covered the objective of breed-genetic characterization by means of the within-breed diversity study, the second purpose of this study was to explore Macabea phylogenetic evolution. This second objective was tackled by means of the between-breeds diversity study, taking into account representatives of all branches used as outgroups, representing different hypothesis of the origin, genetic relationships, and evolution of the Macabea breed. The results of the Reynolds and *F*_ST_ distances were robust: we demonstrated the no existence of zebuines breeds introgression on Macabea, which remark its resistance despite a challenging context with massive introductions of zebuines in the region. In addition, we demonstrated the isolation of Macabea breed with respect of previous introductions of international *Bos taurus* breeds, highly specialized in meat or dairy productions.

Our results on the genetic association of the Macabea with the Spanish breeds, both the southern and northern representatives, are very interesting and support the hypothesis of the traditional integration of the breed in the Shuar-Asuar culture (old “head reducers”). The relationship between the Macabea cattle and the Shuar-Asuar communities, as pointed out by us earlier, probably started in 1599 when the city of Sevilla de Oro; the first European Amazonian settling founded in 1577, was destroyed by these native nationalities [7]. Currently, the Macabea breed displays the best adaptation ability to the wet tropics. However, because of its replacement with exotic zebuines, the breed is highly endangered.

There was a strong association of Macabea with southern Spanish breeds, supporting the hypothesis extracted from the chronicles, which described that the commercial trade between Spain and the American colonies was monopolized by the southern-Spanish ports for a long period [36, 38]. Finally, the closeness of the Macabea with respect to the other creoles, the Colombian HV and BON, included in the study suggests that the origin of the breed would be consistent with the Amazon colonization history, that recall the Spanish expansions from the Pacific area (Figs 2 and 4; S1 Table).

The outcomes from the factorial correspondence analysis support a strong integration of the Macabea with the South American Creoles, grouped with most of the *Bos taurus* members, in particular, when the zebuines were included (Fig 3). When zebuines were excluded, the association among Creoles was magnified. The close proximity between Macabea and the BWS detected in the distance studies (S1 Table) was not supported by the factor correspondence analysis (Fig 3); meanwhile its association with the Spanish BC was reinforced in this analysis. These results support the idea that most of the Creoles originated in areas from the southern Spain, agreeing with previous studies [2, 14].

We used the factorial correspondence analysis to investigate the associations between-breeds based on two different orientations: first, to test the influence of phylogeny on the diversity; and on the another hand, to explore the influence of the original geographical location on the diversity included in the present study. The molecular analysis of variance results suggests that both the phylogenetic origin and the geographical expansions determined the formation of the current diversity; however, the phylogenetic influence is stronger than the geographical evolution, at least in the present context.

Neighbor-joining representation, based on Reynolds distances, is also much illustrative. In Fig 4, *Bos taurus* and *Bos indicus* branches resulted clearly separated, showing that the admixture between both original branches is still not generalized, despite the current zebuine expansion across the continent. The definition of the Macabea as a homogenous breed, its geo-evolutionary relation with other Creoles, and the association in its origin with the BC are also supported by these findings. Macabea breed was isolated in respect to the representatives of the European cosmopolitan breeds and zebuines, which currently represent the most important of the Creole genetic erosive effects [2]. In other words, the Amazonian isolation in which deep-jungle native communities are located, have acted as a protection to the extended indiscriminant and anarchical crossbreeding of cattle in the region.

In addition, we obtained conclusive outcomes from the structure analysis, taking into account that this technique evaluates the level of admixture among individuals from a population based on the individual genetic composition; thus, based on the within-variability, estimates different levels of influence by other populations. In this analysis, as part of the expected likely value of K around the number of breeds included (S1 Fig), we obtained a higher peak at K = 3, which could be interpreted as the effect of the strong differentiation introduced with the zebuine breed types. A first cluster was formed by the Macabea, HV, and BC breeds, thus, supports previous findings. Here, we did not register any other significant influences on the Macabea breed. In addition, we detected a clear sub-structure within the Macabea population, consisting in two subpopulations. This should be considered for the development of a genetic management plan that aims to maintain the maximum level of genetic diversity.

Finally, the representation of the interpolation of admixture coefficients (Fig 6) supports the hypothesis of a cattle expansion from the Pacific areas through the Amazon region, consequently originating the Macabea breed.

## Conclusions

Macabea breed presented molecular genetics parameters that demonstrated genetic stability, typical of differentiated breeds. This finding, together with its position in all the between-population diversity tests developed, supports the suggestion of the Macabea being an important breed integrated as an Ecuadorian zoogenetic patrimony, in particular for the Shuar-Asuar nationalities. These outcomes justify an urgent call for action to the authorities, NGOs, or the private sector to recognize, protect and to valuate these animals and their products.

In the present study, we demonstrated that the Macabea breed originated from Spanish cattle populations located in the Pacific coastal regions of Colombia and Ecuador during the early period of colonization. These cattle populations reached the Amazon along with the first Spanish attempts of colonization and were further introduced in the local native nationalities during the invasion and interactions that occurred at the end of the 16th century. Nevertheless, these evidences need to be reinforced in the future by means of Y-Chromosome and mitochondrial marker studies.

Macabea breed showed a high level of purity; thus, until now, it has avoided the strong influences of modern exotic breeds that have extended across the region, in particular the zebuines. Finally, we need to highlight the importance of the sub-structure detected in the Macabea. In a population without migrations, this sub structure is a positive finding, because it reflects high level of genetic diversity, which must be considered in further conservation programs.

## Supporting Information

S1 Fig Delta K plot following the Evano et al (2005) method, indicating the most likely number of K in Structure analysis software. (PDF)

S1 Table Pairwise Genetic Distances between Populations According to the Model of Reynolds (1983) (Upper Diagonal) and *F*_ST_ (Lower Diagonal). (DOCX)

## References

[1] Delgado JV, Martínez AM, Acosta A, Alvarez LA, Armstrong E, Camacho E, et al Genetic characterization of Latin-American Creole cattle using microsatellite markers. Animal Genetics. 2012;43(1):2–10. 10.1111/j.1365-2052.2011.02207.x 22221019

[2] Martinez AM, Gama LT, Canon J, Ginja C, Delgado JV, Dunner S, et al Genetic footprints of Iberian cattle in America 500 years after the arrival of Columbus. PLoS One. 2012;7(11):e49066 Epub 2012/11/17. 10.1371/journal.pone.0049066 PONE-D-12-01789 [pii]. 23155451 PMC3498335

[3] Primo AT. El ganado bovino Ibérico en las Americas: 500 años después. (The Iberic cattle in the Americas: 500 years later). Archivos de Zootecnia. 1992;41:421–32.

[4] Villalobos-Cortés A, Martínez A, Vega-Pla JL, Landi V, Quiroz J, Marques JR, et al Genetic Relationships Among Five Zebu Breeds Naturalized in America Accessed with Molecular Markers. Italian Journal of Animal Science. 2015;14(2):3280 10.4081/ijas.2015.3280

[5] Clutton-Brock J. A natural history of domesticated mammals. Cambridge: Cambridge University Press; 1999.

[6] Bradley DG, Loftus RT, Cunningham P, MacHugh DE. Genetics and domestic cattle origins. Evolutionary Anthropology: Issues, News, and Reviews. 1998;6(3):79–86. 10.1002/(SICI)1520-6505(1998)6:3<79::AID-EVAN2>3.0.CO;2-R

[7] Rosset E. La conquista del Amazonas: La increíble odisea de la expedición de Orellana. Conocido M, editor: Hondarribia; 2000.

[8] FAO. The state of the world's animal genetic resources for food and agriculture. Rome: 2007.

[9] Ceccobelli S, Di Lorenzo P, Lancioni H, Castellini C, Monteagudo Ibáñez LV, Sabbioni A, et al Phylogeny, genetic relationships and population structure of five Italian local chicken breeds. 2013 2013;12(3). Epub 2013-07-09. 10.4081/ijas.2013.e66

[10] Salamon D, Gutierrez-Gil B, Arranz JJ, Barreta J, Batinic V, Dzidic A. Genetic diversity and differentiation of 12 eastern Adriatic and western Dinaric native sheep breeds using microsatellites. Animal. 2014;8(2):200–7. Epub 2014/01/18. S1751731113002243 [pii] 10.1017/S1751731113002243 . 24433957

[11] Martin-Burriel I, Rodellar C, Canon J, Cortes O, Dunner S, Landi V, et al Genetic diversity, structure, and breed relationships in Iberian cattle. J Anim Sci. 2011;89(4):893–906. Epub 2011/03/19. 89/4/893 [pii] 10.2527/jas.2010-3338 . 21415418

[12] Abdul-Muneer PM. Application of microsatellite markers in conservation genetics and fisheries management: recent advances in population structure analysis and conservation strategies. Genet Res Int. 2014;2014:691759 Epub 2014/05/09. 10.1155/2014/691759 24808959 PMC3997932

[13] Tapio I, Varv S, Bennewitz J, Maleviciute J, Fimland E, Grislis Z, et al Prioritization for conservation of northern European cattle breeds based on analysis of microsatellite data. Conservation Biology. 2006;20(6):1768–79. 10.1111/j.1523-1739.2006.00488.x 17181812

[14] Ginja C, Telo Da Gama L, Penedo MC. Analysis of STR markers reveals high genetic structure in Portuguese native cattle. J Hered. 2010;101(2):201–10. Epub 2009/12/08. esp104 [pii] 10.1093/jhered/esp104 . 19965912

[15] FAO. Secondary guidelines for development of national farm animal genetic resources plans. Roma: FAO, 2004.

[16] Park SDE. Trypanotolerance in West African Cattle and the Population Genetic Effects of Selection Dublin: University of Dublin; 2001.

[17] Belkhir K, Borsa P, Chikhi L, Raufastey N, Bonhomme F. Genetix: 4.05 Logiciel sous WindowsTM pour la genetique des populations. In: Montpellier U. d. (ed.) Montpellier, France 2003.

[18] Raymond M, Rousset F. GENEPOP (Version 1.2): Population genetics software for exact test and ecumenicism. Journal of Heredity. 1995;86(3):248–9.

[19] Guo SW, Thompson EA. Performing the exact test of Hardy-Weinberg proportions for multiple alleles. Biometrics. 1992;48:361–72. 1637966

[20] Reynolds J, Weir BS, Cockerham CC. Estimation of the coancestry coefficient: basis for a short-term genetic distance. Genetics. 1983;105:767–79. 17246175 10.1093/genetics/105.3.767 PMC1202185

[21] Langella O. Population Genetic Software (Individuals or Populations Distances, Phylogenetic Trees) 1999 [updated 12/5/2002]. Available: http://www.bioinformatics.org/download.php?fileid=430.

[22] Excoffier L, Lischer HE. Arlequin suite ver 3.5: a new series of programs to perform population genetics analyses under Linux and Windows. Molecular Ecology Resources. 2010;10(3):564–7. Epub 2011/05/14. 10.1111/j.1755-0998.2010.02847.x . 21565059

[23] Huson DH, Bryant D. Application of phylogenetic networks in evolutionary studies. Mol Biol Evol. 2006;23:254–67. 10.1093/molbev/msj030 16221896

[24] Pritchard JK, Stephens M, Donnelly P. Inference of Population Structure Using Multilocus Genotype Data. Genetics. 2000;155(2):945–59. 10835412 10.1093/genetics/155.2.945 PMC1461096

[25] Rosenberg NA. Distruct: a program for the graphical display of structure results. Available: http://www.cmb.usc.edu/»noahr/distruct.html. 2002.

[26] Evanno G, Regnaut S, Goudet J. Detecting the number of clusters of individuals using the software STRUCTURE: a simulation study. Molecular ecology. 2005;14(8):2611–20. 10.1111/j.1365-294X.2005.02553.x . 15969739

[27] Jay F, Manel S, Alvarez N, Durand EY, Thuiller W, Holderegger R, et al Forecasting changes in population genetic structure of alpine plants in response to global warming. Molecular ecology. 2012;21(10):2354–68. 10.1111/j.1365-294X.2012.05541.x . 22512785

[28] R Core Team. R: A Language and Environment for Statistical Computing. 2012.

[29] Canon J, Alexandrino P, Bessa I, Carleos C, Carretero Y, Dunner S, et al Genetic diversity measures of local European beef cattle breeds for conservation purposes. Genet Sel Evol. 2001;33:311–32. 10.1051/gse:2001121 11403750 PMC2705410

[30] Weir BS. Genetic Data Analysis. 1990.

[31] Weir BS, Cockerham CC. Estimating F statistics for the analysis of population structure. Evolution. 1984;38:1358–70. 28563791 10.1111/j.1558-5646.1984.tb05657.x

[32] Cañón J, García D, Delgado JV, Dunner S, Telo da Gama L, Landi V, et al Relative breed contributions to neutral genetic diversity of a comprehensive representation of Iberian native cattle. Animal. 2011;5(9):1323–34. 10.1017/S1751731111000267 22440277

[33] Mateus JC, Penedo MCT, Alves VC, Ramos M, Rangel-Figueiredo T. Genetic diversity and differentiation in Portuguese cattle breeds using microsatellites. Animal Genetic. 2004;35:106–13. 10.1111/j.1365-2052.2004.01089.x 15025569

[34] Liron JP, Peral-Garcia P, Giovambattista G. Genetic characterization of Argentine and Bolivian Creole cattle breeds assessed through microsatellites. Journal of Heredity. 2006;97(4):331–9. Epub 2006/06/24. esl003 [pii] 10.1093/jhered/esl003 . 16793865

[35] Egito A, Paiva S, Albuquerque MdS, Mariante A, Almeida L, Castro S, et al Microsatellite based genetic diversity and relationships among ten Creole and commercial cattle breeds raised in Brazil. BMC Genetics. 2007;8(1):83 10.1186/1471-2156-8-83 18067665 PMC2228320

[36] Villalobos Cortés AI, Martínez AM, Escobar C, Vega-Pla JL, Delgado JV. Study of genetic diversity of the Guaymi and Guabala bovine populations by means of microsatellites. Livestock Science. 2010;131 45–51.

[37] FAO. Secondary Guidelines for Developmen of National Farm Animal Genetic Resources Management Plans: Managemen of small populations at risk. Rome. Italy: FAO, 2005.

[38] Rodero A, Delgado JV, Rodero E. Primitive andalusian livestock an their implications in the discovery of America. Archivos de Zootecnia. 1992;41 (Extra):383–400.

