# Supplementary figures and images for: Molecular Study of the Amazonian Macabea Cattle History
Source: PLoS One. 2016 Oct 24;11(10):e0165398. doi: 10.1371/journal.pone.0165398 (PMC5077120; doi:10.1371/journal.pone.0165398)

$$\text{DeltaK} = \text{mean}(|L''(K)|) / \text{sd}(L(K))$$

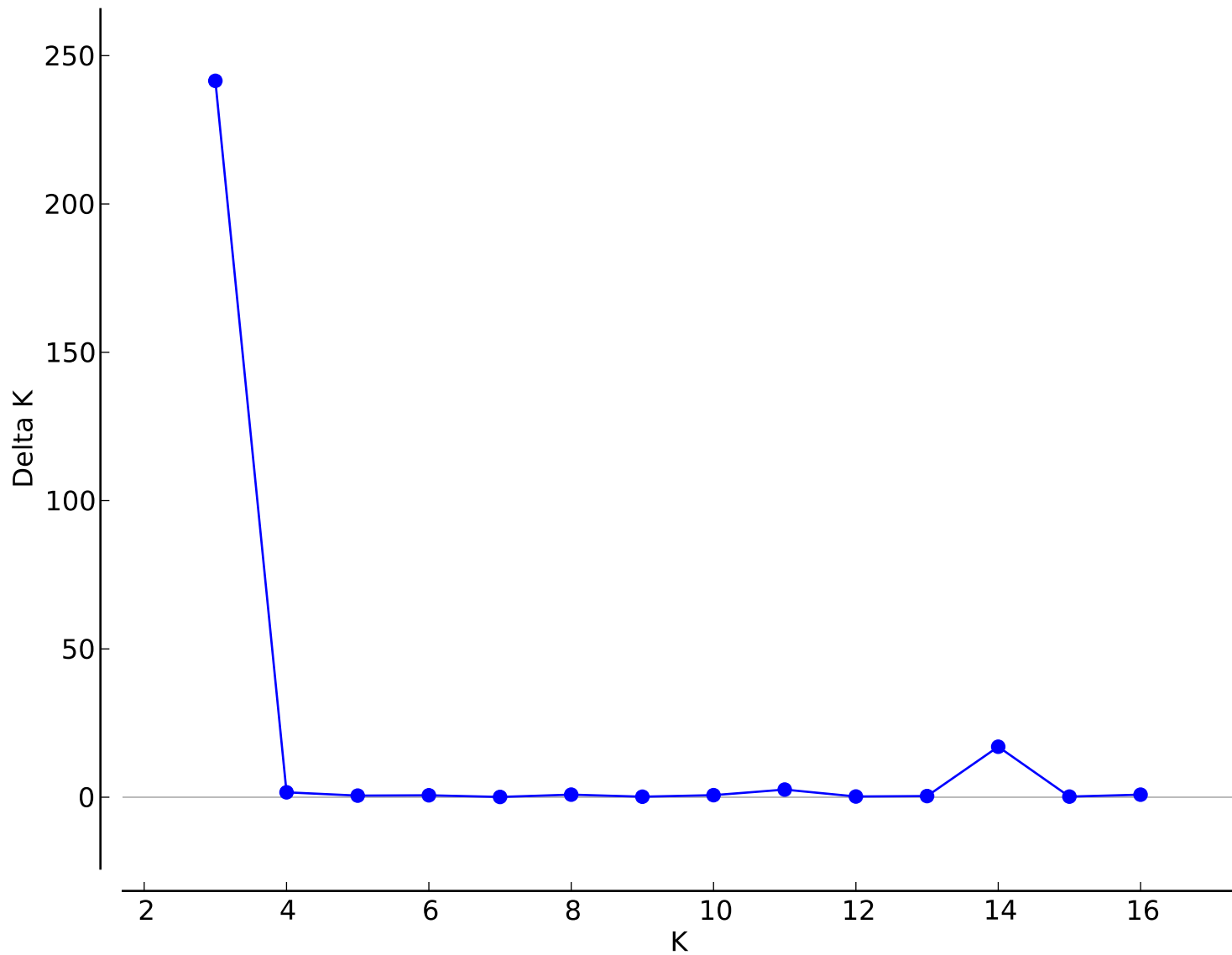

Supplement: S1 Fig — (PDF) [file pone.0165398.s001.pdf]
