# Supplementary material for: Molecular Study of the Amazonian Macabea Cattle History
Source: PLoS One. 2016 Oct 24;11(10):e0165398. doi: 10.1371/journal.pone.0165398 (PMC5077120; doi:10.1371/journal.pone.0165398)
Supplement: S1 Table — (DOCX) [file pone.0165398.s002.docx]

**Table S3. Pairwise Genetic Distances between Populations According to the Model of Reynolds (1983) (Upper Diagonal) and *F*_ST_ (Lower Diagonal).**

|  | **BON** | **HV** | **EC** | **RGA** | **BC** | **MA** | **HER** | **JER** | **BWS** | **CHAR** | **FRI** | **SIM** | **GYR** | **BRH** | **NEL** | **ZEBU** | **MAC** |
| --- | --- | --- | --- | --- | --- | --- | --- | --- | --- | --- | --- | --- | --- | --- | --- | --- | --- |
| **BON** |  | 0.04 | 0.07 | 0.10 | 0.05 | 0.08 | 0.13 | 0.14 | 0.10 | 0.10 | 0.08 | 0.11 | 0.26 | 0.22 | 0.26 | 0.22 | 0.07 |
| **HV** | 0.04 |  | 0.02 | 0.07 | 0.02 | 0.04 | 0.08 | 0.11 | 0.05 | 0.06 | 0.04 | 0.06 | 0.19 | 0.14 | 0.18 | 0.15 | 0.04 |
| **EC** | 0.07 | 0.01 |  | 0.07 | 0.03 | 0.05 | 0.08 | 0.12 | 0.06 | 0.07 | 0.05 | 0.07 | 0.16 | 0.13 | 0.17 | 0.14 | 0.04 |
| **RGA** | 0.1 | 0.06 | 0.07 |  | 0.06 | 0.08 | 0.11 | 0.13 | 0.08 | 0.07 | 0.09 | 0.08 | 0.26 | 0.21 | 0.27 | 0.20 | 0.11 |
| **BC** | 0.05 | 0.02 | 0.03 | 0.05 |  | 0.02 | 0.08 | 0.09 | 0.05 | 0.05 | 0.06 | 0.07 | 0.20 | 0.16 | 0.20 | 0.16 | 0.06 |
| **MA** | 0.07 | 0.03 | 0.05 | 0.08 | 0.03 |  | 0.10 | 0.13 | 0.06 | 0.07 | 0.08 | 0.08 | 0.22 | 0.18 | 0.22 | 0.20 | 0.08 |
| **HER** | 0.13 | 0.08 | 0.07 | 0.10 | 0.08 | 0.09 |  | 0.17 | 0.10 | 0.11 | 0.11 | 0.11 | 0.26 | 0.22 | 0.26 | 0.22 | 0.11 |
| **JER** | 0.13 | 0.11 | 0.12 | 0.12 | 0.09 | 0.13 | 0.16 |  | 0.15 | 0.11 | 0.13 | 0.15 | 0.31 | 0.24 | 0.28 | 0.22 | 0.15 |
| **BWS** | 0.09 | 0.04 | 0.05 | 0.07 | 0.04 | 0.05 | 0.09 | 0.14 |  | 0.08 | 0.07 | 0.08 | 0.25 | 0.21 | 0.26 | 0.22 | 0.07 |
| **CHAR** | 0.09 | 0.05 | 0.07 | 0.07 | 0.05 | 0.06 | 0.10 | 0.10 | 0.07 |  | 0.09 | 0.06 | 0.27 | 0.23 | 0.27 | 0.22 | 0.10 |
| **FRI** | 0.08 | 0.04 | 0.05 | 0.08 | 0.05 | 0.07 | 0.10 | 0.13 | 0.06 | 0.08 |  | 0.09 | 0.27 | 0.23 | 0.27 | 0.21 | 0.08 |
| **SIM** | 0.10 | 0.06 | 0.07 | 0.08 | 0.07 | 0.08 | 0.10 | 0.15 | 0.08 | 0.06 | 0.08 |  | 0.27 | 0.22 | 0.28 | 0.23 | 0.09 |
| **GYR** | 0.23 | 0.16 | 0.14 | 0.21 | 0.17 | 0.19 | 0.21 | 0.27 | 0.22 | 0.23 | 0.22 | 0.24 |  | 0.04 | 0.10 | 0.13 | 0.23 |
| **BRH** | 0.21 | 0.13 | 0.12 | 0.19 | 0.15 | 0.17 | 0.20 | 0.23 | 0.19 | 0.21 | 0.20 | 0.21 | 0.03 |  | 0.06 | 0.10 | 0.19 |
| **NEL** | 0.24 | 0.16 | 0.16 | 0.23 | 0.19 | 0.20 | 0.23 | 0.26 | 0.22 | 0.24 | 0.24 | 0.25 | 0.09 | 0.06 |  | 0.14 | 0.23 |
| **ZEBU** | 0.20 | 0.14 | 0.14 | 0.19 | 0.15 | 0.18 | 0.20 | 0.20 | 0.20 | 0.21 | 0.20 | 0.22 | 0.11 | 0.10 | 0.13 |  | 0.18 |
| **MAC** | 0.07 | 0.04 | 0.04 | 0.10 | 0.06 | 0.08 | 0.11 | 0.14 | 0.06 | 0.10 | 0.08 | 0.09 | 0.20 | 0.18 | 0.21 | 0.18 |  |

BON: Blanco Orejinegro; HV: Hartón del Valle; EC: Ecuadorian Creole; RGA: Rubia Gallega; BC: Berrenda en Colorado; MA: Marismeña; HER: Hereford; JER: Jersey; BWS: Brown Swiss; CHAR: Charolais; FRI: Holstein Friesian; SIM: Simmental; ZEBU, Zebu; NE: Nelore; BRH: Brahman; GYR: Gyr; MAC: Macabea.
